# Sparse Computation in Adaptive Spiking Neural Networks

**DOI:** 10.3389/fnins.2018.00987

**Published:** 2019-01-08

**Authors:** Davide Zambrano, Roeland Nusselder, H. Steven Scholte, Sander M. Bohté

**Affiliations:** ^1^Machine Learning Group, CWI, Amsterdam, Netherlands; ^2^Programme Group Brain and Cognition, Faculty of Social and Behavioural Sciences, University of Amsterdam, Amsterdam, Netherlands; ^3^Faculty of Sciences, Swammerdam Institute for Life Sciences, University of Amsterdam, Amsterdam, Netherlands

**Keywords:** spiking neural networks, neural coding, adaptive spiking neurons, attention, deep neural networks

## Abstract

Artificial Neural Networks (ANNs) are bio-inspired models of neural computation that have proven highly effective. Still, ANNs lack a natural notion of time, and neural units in ANNs exchange analog values in a frame-based manner, a computationally and energetically inefficient form of communication. This contrasts sharply with biological neurons that communicate sparingly and efficiently using isomorphic binary spikes. While Spiking Neural Networks (SNNs) can be constructed by replacing the units of an ANN with spiking neurons (Cao et al., 2015; Diehl et al., 2015) to obtain reasonable performance, these SNNs use Poisson spiking mechanisms with exceedingly high firing rates compared to their biological counterparts. Here we show how spiking neurons that employ a form of neural coding can be used to construct SNNs that match high-performance ANNs and match or exceed state-of-the-art in SNNs on important benchmarks, while requiring firing rates compatible with biological findings. For this, we use spike-based coding based on the firing rate limiting adaptation phenomenon observed in biological spiking neurons. This phenomenon can be captured in fast adapting spiking neuron models, for which we derive the effective transfer function. Neural units in ANNs trained with this transfer function can be substituted directly with adaptive spiking neurons, and the resulting Adaptive SNNs (AdSNNs) can carry out competitive classification in deep neural networks without further modifications. Adaptive spike-based coding additionally allows for the dynamic control of neural coding precision: we show empirically how a simple model of arousal in AdSNNs further halves the average required firing rate and this notion naturally extends to other forms of attention as studied in neuroscience. AdSNNs thus hold promise as a novel and sparsely active model for neural computation that naturally fits to temporally continuous and asynchronous applications.

## Introduction

With rapid advances in deep neural networks, renewed consideration is given to the question how artificial neural networks relate to the details of information processing in real biological *spiking* neurons. Apart from its still vastly more flexible operation, the huge spiking neural network that comprises the brain is also highly energy efficient. This derives in large part from its sparse activity: estimates are that neurons in mammalian brains on average only emit somewhere between 0.2 and 5 spikes per second (Attwell and Laughlin, 2001). In contrast, current best-performing deep neural networks using spiking neurons—spiking neural networks (SNNs)—use stochastic Poisson neurons with exceedingly high firing rates, up to hundreds of Hertz on average, to cover the dynamic range of corresponding analog neurons (Cao et al., 2015; Diehl et al., 2015).

In biology, sensory neurons adaptively control the number of spikes that are used to efficiently cover large dynamic ranges (Fairhall et al., 2001). This adaptive behavior can be captured with fast (< 100ms) spike frequency adaptation in Leaky-Integrate-and-Fire neuron models, or corresponding Spike Response Models (SRMs) (Gerstner and Kistler, 2002; Bohte, 2012; Pozzorini et al., 2013) including the Adaptive Spiking Neuron models (ASN) (Bohte, 2012). ASNs can implement adaptive spike-based coding as a neural coding scheme that maps analog values to sequences of spikes, where the thresholding mechanism carries out an online analog-to-digital conversion of the analog signal computed in the neuron unit. Here, we demonstrate the effectiveness of such neurons to create powerful deep SNNs.

With adaptive spike-based coding, the neural coding precision can be dynamically modulated in a straightforward manner. We show how the tuneable relationship between firing rate and neural coding precision can be exploited to further lower the average firing rate by selectively manipulating this trade off as a particular form of attention. It is well known that for stable sensory inputs, neural correlates of attention in the brain include enhanced firing in affected neurons (Roelfsema et al., 1998). One purported effect of this mechanism is to improve neural coding precision on demand, for instance in specific locations, for a brief amount of time, and only if needed (Friston, 2010; Saproo and Serences, 2010). Such attention would allow the brain to process information at a low default precision when possible and increase firing rate only when necessary, potentially saving a large amount of energy.

### Adaptive Spike-Based Coding

**Figure 1:**
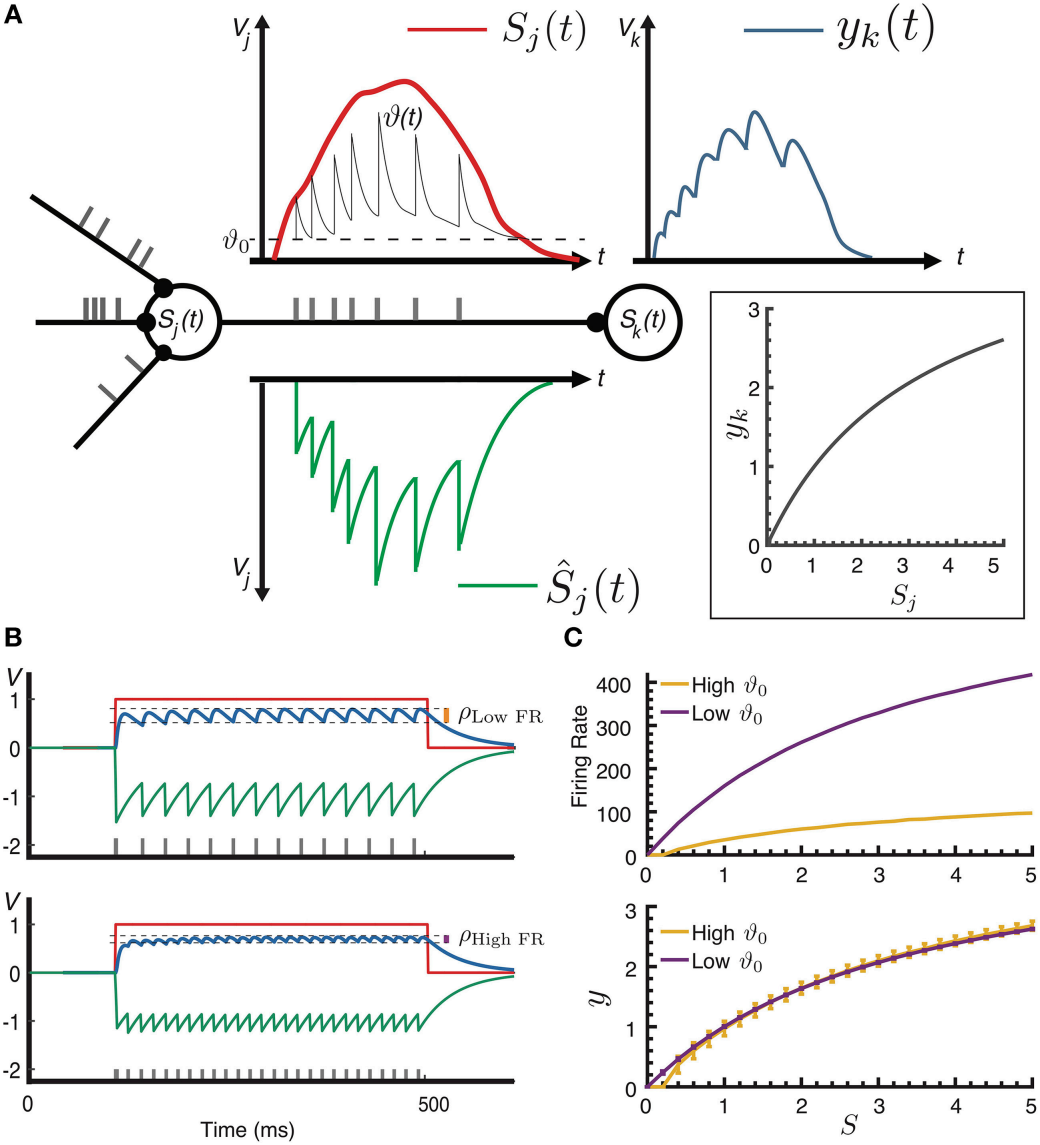
**(A)** Adaptive spike-based coding. In a neuron *j*, spikes (gray ticks) are generated when the difference between the activation *S*_*j*_(*t*)(red line, measured in potential *V*_*j*_) and the refractory response Ŝ_*j*_(*t*) (green line) exceeds the dynamic threshold ϑ(*t*). Emitted spikes contribute a sum of PSPs to the target neuron's potential *y*_*k*_(*t*) (blue line). Inset: effective transfer function *y*_*k*_ = *f*(*S*_*j*_). **(B)** Example of encoding a step-function activation at two different neural coding precisions: the resultant normalized postsynaptic contribution *y*(*t*) (blue line) is plotted for two different values of the resting threshold ϑ_0_ = [0.25, 0.05]. Compared to the neural coding precision ρ_Low FR_ at ϑ_0_ = 0.25, the lower threshold ϑ_0_ = 0.05 results in a higher firing rate and a higher neural precision (ρ_High FR_). **(C)** Top: two values of ϑ_0_ result in two different firing rate curves; bottom: the effective transfer function for two values of ϑ_0_, the same approximated value can be represented with different precisions (measured as deviation from the average), and thus different firing rates, by controlling the effective spike height *h*.

Adaptive spike-based coding is illustrated in Figure 1A: expressed as an SRM, the membrane potential *V* of a neuron *j* is computed as the difference between input *S*(*t*), and the refractory response Ŝ(*t*) that models the hyper-polarization of the membrane potential upon spike emission. The input here is a sum of postsynaptic potentials (PSP) due to spikes from presynaptic input neurons *i* impinging at times *t*_*i*_ weighted by synaptic efficacy *w*_*ij*_ plus any injected input *V*_inj, *j*_(*t*), and the refractory response is modeled as a sum of scaled spike-triggered refractory kernels η(*t*):

$$\begin{array}{l} V_{j}(t)=S_{j}(t)-{\hat{S}}_{j}(t)=\left[V_{\text{inj},j}(t)+\sum_{i}\sum_{t_{i}}w_{ij}h\kappa (t-t_{i})\right]\\ \;-\sum_{j}\sum_{t_{j}}\vartheta (t_{j})\eta (t-t_{j}), \end{array}$$

where spikes are emitted at times *t*_*j*_ when the potential exceeds a dynamic threshold ϑ(*t*), the PSP is modeled as a normalized kernel κ(*t* − *t*_*i*_) with height *h*, the effective spike height, and the refractory kernel η(*t*) is adaptively scaled with the threshold at the time of firing; importantly, Ŝ_*j*_(*t*) thus approximates the rectified activation ${\left[S_{j}\left(t\right)\right]}^{+}$. For a fixed threshold, that is without adaptation, the model corresponds to an SRM_0_ formulation of the Leaky-Integrate-and-Fire neuron (Gerstner and Kistler, 2002)[Section 4.2.3] using the Asynchronous Pulsed Sigma-Delta Modulation (APSDM) scheme as noted by Yoon (2016). In practical terms, without adaptation the threshold and weights need to be tuned to the dynamic range of the spiking neuron, as was done for instance in Diehl et al. (2015). Following (Bohte, 2012; Pozzorini et al., 2013), spike frequency adaptation is incorporated into the model by multiplicatively increasing the variable threshold ϑ(*t*) at the time of spiking with a decaying adaptation kernel γ(*t*):

(1) $$\begin{array}{l} \vartheta_{j}\left(t\right)=\vartheta_{0}+\underset{t_{j}}{\sum }\frac{m_{f}}{\vartheta_{0}}\vartheta \left(t_{j}\right)\gamma \left(t-t_{j}\right), \end{array}$$

where ϑ_0_ is the resting threshold and the multiplicative parameter *m*_*f*_ controls the speed of the adaptation. Both η(*t*) and γ(*t*) kernels affect the neuron's spike frequency adaptation; intuitively, the refractory response relates to the amount of signal that is communicated to the downstream target neurons, while the dynamic threshold adapts the neurons response to changes in the dynamic range of its input.

In the terminology of Gerstner and Kistler (2002), the proposed adaptive spike-based coding constitutes a variant of rate-coding, where the rate in measured not in terms of the spike-interval or an average population activity, but rather as the effective sum of PSPs on the target neuron. For a dynamic input current, the timing of individual spikes allows this postsynaptic sum to track this signal (Bohte, 2012); for a fixed input current, the adaptive spiking mechanism effectively maps an activation *S*_*j*_ to a normalized average contribution *y*(*S*_*j*_) to the next neuron's activation *S*_*k*_ as a rectified half-sigmoid-like transfer function (Figure 1A, inset):

(2) $$y_{j}=f(S_{j})=\;\langle \sum_{t_{j}}\kappa (t-t_{j})\rangle ,$$

and we can derive an analytical expression for the shape of the transfer function *f* (*S*) to map spiking neurons to analog neural units (see section Materials and Methods). The use of exponentially decaying kernels for η(*t*), γ(*t*) and κ(*t*) allows the neuron model to be efficiently computed with simple dynamical systems.

The speed of adaptation *m*_*f*_ and the effective spike height *h* together control the precision of the spike-based neural coding, where the spiking neuron's neural coding precision is measured as the deviation of *y*(*t*) from the mean response to a fixed input for the spiking neuron. As illustrated in Figure 1B, a same-but-more-precise spike-based encoding can be realized by changing the adaptation parameters *m*_*f*_, ϑ_0_ to increase the firing rate for a given stimulus intensity, while simultaneously reducing the impact of spikes on target neurons by decreasing *h* (corresponding to a global reduction of synaptic efficacy). An ASN can thus map different stimulus-to-firing-rate curves (Figure 1C, top) to the same transfer function but with different neural coding precision (Figure 1C, bottom). We exploit this ability to encode the same signal with different precisions in a toy model of attention, where neural coding precision is increased in the entire network as a form of Arousal.

## Materials and Methods

### Adaptive Spiking Neurons

In the ASN, the PSP kernel κ(*t*) is computed as the convolution of a spike-triggered postsynaptic current (PSC) with a filter ϕ, with the PSC decaying exponentially with time constants τ_ϕ_ and the filter ϕ decaying with time-constant τ_β_; an injected input *V*_inj,*j*_(*t*) is similarly computed from a current injection *I*_inj,*j*_(*t*). The adaptation kernel γ(*t*) decays with time-constant τ_γ_.

The AdSNNs's are created by converting standard Deep Neural Networks (Diehl et al., 2015) trained with a mathematically derived transfer function *f*(*S*) of the ASN (full derivation in Supplementary Material), defined as the function that maps the activation *S* to the average post-synaptic contribution. This has the form:

$$\begin{array}{l} f\left(S\right)=\max\left(0,\frac{h}{\exp\left(\frac{c_{1}\cdot S+c_{2}}{c_{3}\cdot S+c_{4}}\right)-1}-c_{0}+h/2\right), \end{array}$$

where,

$$\begin{array}{l} c_{1}=2\cdot \frac{m_{f}}{\vartheta_{0}}\cdot \tau_{\gamma }^{2},\\ c_{2}=2\cdot \vartheta_{0}\cdot \tau_{\eta }\cdot \tau_{\gamma },\\ c_{3}=\tau_{\gamma }\cdot \left(\frac{m_{f}}{\vartheta_{0}}\cdot \tau_{\gamma }+\left(2\cdot \frac{m_{f}}{\vartheta_{0}}+1\right)\cdot \tau_{\eta }\right),\\ c_{4}=\vartheta_{0}\cdot \tau_{\eta }\cdot \left(\tau_{\gamma }+\tau_{\eta }\right),\\ c_{0}=\frac{h}{\exp\left(\frac{c1\cdot \vartheta_{0}+c2}{c3\cdot \vartheta_{0}+c4}\right)-1}, \end{array}$$

are constants computed from the neuron parameters setting, and *h* defines the spike size. Here, by normalizing *f*(*S*) to 1 when *S* = 1, *h* becomes a scaling factor for the network's trained weights, allowing communication with binary spikes.

### Adaptive Spiking Neural Networks (AdSNNs)

**Figure 2:**
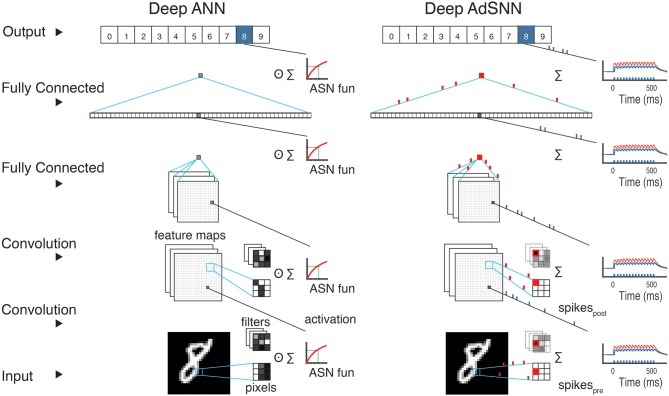
Adaptive Spiking Neural Network conversion schematic. **(Left)** During training of a deep ANN, the output of a convolutional or fully connected layer is passed through the ASN transfer function. In ANNs, every layer performs a series of weighted sums of the inputs as each analog input is multiplied by its analog weight. **(Right)** For classification, the analog ANN units are converted in ASNs to obtain an SNN. In each layer, the signal *S* (red line) is collected and then converted to spikes. All the network layers perform similarly, but with ASNs binary spikes are conveyed across the network and corresponding weights are simply added to membrane-current *I* of the ASN in the next layer.

Analog units using *f*(*S*) as their transfer function, which we denote as Adaptive Artificial Neurons– **AANs**, in trained ANNs can be replaced directly and without modification with ASNs (see Figure 2). In the presented results, the adaptation kernel γ(*t*) decays with τ_γ_ = 15ms, the membrane filter ϕ(*t*) with τ_ϕ_ = 5ms, the refractory response η(*t*) with τ_η_ = 50ms and the PSC with τ_β_ = 50ms, all compatible with the range of values observed in biological neurons (Gerstner et al., 2014) [Section 3.1], and $m_{f}=\vartheta_{0}^{2}$. Batch Normalization (BN) (Ioffe and Szegedy, 2015) is used to avoid the vanishing gradient problem (Hochreiter, 1998) for saturating transfer functions like half-sigmoids and to improve the network training and regularization. After training, the BN layers are removed and integrated into the weights' computation (Rueckauer et al., 2017). A BN-AAN layer is also used as a first layer in all the networks to convert the inputs into spikes. When converting, biases are added to the post-synaptic activation. Max and Average Pooling layers are converted by merging them into the next ASN-layer: the layer activation *S* is computed from incoming spikes, then the pooling operator is applied and the ASN-layer computes spikes as output. The last ASN layer acts as a smoothed read-out layer with τ_ϕ_ = 50ms, where spikes are converted into analog values for classification. The classification is performed as in the ANN network, usually using SoftMax: at every time-step *t* the output with highest value is considered the result of the classification.

### ANN Training

We trained ANN with AANs on widely used datasets: for feedforward ANNs, IRIS and SONAR; and for deep convolutional ANNs: MNIST, CIFAR-10, CIFAR-100 and ILSVRC-2012. All the ANNs are trained using Keras^1^ with Tensorflow^2^ as its backend. We used categorical cross-entropy as a loss function with Adam (Kingma and Ba, 2014) as the optimizer, except for ILSVRC-2012 where we used Stochastic Gradient Decent with Nesterov (learning rate = 1*e*−3, decay = 1*e*−4 and momentum = 0.9). Since we aim to convert high performance ANNs into AdSNNs, for each dataset, we selected the model at the training epoch where it performed best on the test set.

We trained a [4−60−60−3] feedforward ANN on the IRIS dataset: IRIS is a classical non-linearly separable toy dataset containing 3 classes—3 types of plants—with 50 instances each, to be classified from 4 input attributes. Similarly, for the SONAR dataset (Gorman and Sejnowski, 1988) we used a [60−50−50−2] ANN to classify 208 entries of sonar signals divided in 60 energy measurements in a particular frequency band in two classes: metal cylinder or simple rocks. We trained both ANNs for 800 epochs and obtained competitive performance.

The deep convolutional ANNs are trained on standard image classification problems with incremental difficulty. The simplest is the MNIST dataset (Lecun et al., 1998), where 28 × 28 images of handwritten digits have to be classified. We used a convolutional ANNs composed of [28×28 -− *c*64×3 -− *m*2 -− 2×(*c*128×3 -− *c*) -− *m*2 -− *d*256 -− *d*50 -− 10], where *cN*×*M* is a convolutional layer with *N* feature maps and a kernel size of *M*×*M*, *mP* is a max pooling layer with kernel size *P*×*P*, and *dK* is a dense layer with *K* neurons. Images are pre-normalized between 0 and 1, and the convolutional ANN was trained for 50 epochs. We found that using average pooling gives slightly worse performance, as typically reported; and max pooling could be implemented in biology as a multi-compartment neuron (Larkum et al., 2009).

The CIFAR-10 and CIFAR-100 data sets (Krizhevsky, 2009) are harder benchmarks, where 32×32 color images have to be classified in 10 or 100 categories respectively. We use a VGG-like architecture (Simonyan and Zisserman, 2014) with 12 layers: [32×32 -− 2×(*c*64×3) -− *m*2 -− 2×(*c*128×3) -− *m*2 -− 3×(*c*256×3) -− *m*2 -− 3×(*c*512×3) -− *m*2 -− *d*512 -− 10]for CIFAR-10 and [32×32 -− 2×(*c*64×3) -− *m*2 -− 2×(*c*128×3) -− *m*2 -− 3×(*c*256×3) -− *m*2 -− 3×(*c*1024×3) -− *m*2 -− *d*1024 -− 100] for CIFAR-100. Dropout (Srivastava et al., 2014) was used in the non-pooling layers (0.5 in the top fully-connected layers, and 0.2 for the first 500 epochs and 0.4 for the last 100 in the others). Images were converted from RGB to YCbCr and then normalized between 0 and 1.

The ImageNet Large-Scale Visual Recognition Challenge (ILSVRC) (Russakovsky et al., 2015) is a large-scale image classification task with over 15 million labeled high-resolution images belonging to roughly 22, 000 categories. The 2012 task-1 challenge was used, a subset of ImageNet with about 1000 images in each of 1000 categories. We trained a ResNet-18 architecture in the Identity-mapping variant (He et al., 2016) for 100 epochs and the top-1 error rate is reported. As in (Simonyan and Zisserman, 2014), we rescaled the images to a resolution of 256×256 pixels and then performed random cropping during training and centre cropping for testing.

### AdSNN Evaluation

The AdSNNs are evaluated in simulations with 1ms timesteps, where inputs are persistently presented for *T* = [0…500ms] (identical to the method used in Diehl et al., 2015) for IRIS, SONAR and MNIST and for *T* = [0…1, 000ms] for CIFAR-10/100 and ImageNet. The Firing Rate (FR) in Table 1 is computed as the average number of spikes emitted by a neuron, for each image, in this time window. The time window T is chosen such that all output neurons reach a stable value; we defined the Matching Time (MT) as the time in which 101% of the minimum classification error is reached for each simulation^3^. From MT to the end of the time window, the standard deviation of the accuracy is computed to evaluate the stability of the network's response. Each dataset was evaluated for a range of ϑ_0_ values of [0.015, 0.5] and the minimum firing rate needed to match the ANN performance is reported. All the AdSNNs simulations are run on MATLAB in a modified version of the MatConvNet framework^4^.

**Table 1:** Performance (Perf., %), Matching Firing Rate (FR, Hz) and Matching Time (MT, ms).

**DataSet**	**Prev. SNN**	**ANNs (Relu)**	**AdSNNs**			**Arousal AdSNNs**		
	**Perf**.	**Perf**.	**Perf**.	**FR**	**MT**	**Perf**.	**FR**	**MT**
IRIS	–	98.67 (98.7)	**98.67 ± 0.01**	45	269	**98.67 ± 0.01**	**17**	484
SONAR	–	89.42 (89.4)	**89.89 ± 1.15**	25	119	**89.66 ± 0.41**	**11**	414
MNIST	99.12^(1)^	99.59 (99.5)	**99.59 ± 0**	37	350	**99.51 ± 0.01**	**8**	441
CIFAR-10	89.32^(2)^	89.66 (89.9)	**89.67 ± 0.03**	73	424	**89.67 ± 0.04**	**43**	592
CIFAR-100	65.48^(2)^	63.45 (63.5)	63.38 ± 0.06	109	500	63.37 ± 0.08	71	686
ILSVRC-2012	–	62.98 (69.9)	**62.97 ± 0.05**	**97**	347	**62.89 ± 0.28**	**59**	460

*Current SNN performance is compared against trained ANN with ASN transfer function, (with ReLU transfer function), AdSNN and Arousal AdSNNs performance. State of the art is denoted with bold font; no current fully-spiking SNN state of the art exists for IRIS, SONAR or ImageNet. (1): (Diehl et al., 2015) (2): (Esser et al., 2016)*.

### Arousal

The arousal mechanism increases the coding precision in the network by using more spikes with commensurately less postsynaptic impact to convey the same signal value *S*(*t*) to the postsynaptic target with more precision ρ. Arousal is selectively applied only to those samples whose classification is uncertain when processed at a default, lower neural coding precision. The network is simulated with ϑ_0_ set to ϑ_0−*lp*_, the standard low-precision parameter; if the input is selected by the arousal mechanism, the ϑ_0_ parameter is set to the high precision value: ϑ_0−*hp*_ (and *m*_*f*_ and *h* are changed accordingly). Selection is determined by accumulating the winning and the 2nd-highest outputs for 50ms starting from a pre-defined *t*_*sa*_ specific for each dataset. If the difference between these two outputs exceeds a threshold θ_*A*_, the input is not highlighted – θ_*A*_ is estimated by observing those images that are not correctly classified when the precision is decreased on the training set. The ideal Arousal method only selects images that are misclassified at low precision, in the method as defined here, many more images are selected. To quantify this, we defined Selectivity as the proportion of highlighted images (Table **3**) In addition, θ_*A*_ increases linearly with the accumulation time interval as θ_*A*_ = *p*_1_·(*t* − *t*_*sa*_)+*p*_2_, while Selectivity decreases exponentially. We report results for the parameter configuration that resulted in the lowest firing rate on average for each dataset (Figure **4C**), which is obtained at a specific ϑ_0−*lp*_: in fact, starting from very low precision leads to higher Selectivity, which in turn results in a higher average firing rate (Figure S1 reports the final Firing Rates achieved at different ϑ_0−*lp*_ for the MNIST dataset). The parameter ϑ_0−*hp*_ is chosen as the lowest precision needed to match the ANN performance. Table 3 reports the values of Selectivity, *t*_*sa*_, ϑ_0−*lp*_, ϑ_0−*hp*_, *p*_1_, *p*_2_ for each dataset. Note that, since deeper networks need more time to settle to the high precision level, we extended the simulation time for these networks (see Table 1).

## Results

We construct AdSNNs comprised of ASN neurons using adaptive spike-coding similar to the approach pioneered in Diehl et al. (2015) to obtain high performance sparsely active SNNs. First, ANNs are constructed with analog neural units that use the derived half-sigmoid-like transfer function *f* (*S*), both for fully connected feed-forward ANNs and for various deep convolutional neural network architectures. We train these ANNs for standard benchmarks of increasing difficulty (SONAR, IRIS, MNIST, CIFAR-10/100, and the ImageNet Large-Scale Visual Recognition Challenge (ILSVRC 2012) benchmarks). Corresponding AdSNNs are then obtained by replacing the ANNs' analog units with ASNs (illustrated in Figure 2). For comparison, we also trained the identical ANN architectures with ReLU transfer function^5^.

For the biologically compatible spiking neuron parameters used, the AdSNNs match performance to the original ANNs as measured on the test set (Table 1). We trained high-performance ANNs such that the converted AdSNNs exceed previous state-of-the-art performance obtained by fully spiking SNN on almost all benchmarks; the use of the ASN transfer function yields performance equal to the same networks using ReLU's, except for some decline for the ILSVRC dataset. Network sizes, spikes and synaptic operations required for classification are given in Table 2.

**Table 2:** Performance (Perf., %), total number of spikes (NoS) and Synaptic Operations (SOP) to Matching Time (MT, ms), with and without Arousal.

**DataSet**	**Neurons**	**AdSNNs**				**Arousal AdSNNs**			
		**Perf**.	**NoS**	**SOPs**	**MT**	**Perf**.	**NoS**	**SOPs**	**MT**
MNIST	29*K*	99.59	5.4 × 10^5^	5.8 × 10^8^	350	99.51	1.2 × 10^5^	1.6 × 10^8^	441
CIFAR-10	182*K*	89.66	5.7 × 10^6^	6.6 × 10^9^	424	89.67	4.2 × 10^6^	4.7 × 10^9^	592
CIFAR-100	182*K*	63.45	1.9 × 10^7^	1.3 × 10^10^	500	63.37	1.3 × 10^7^	9.2 × 10^9^	686
ILSVRC-2012	1.9*M*	62.98	1.8 × 10^8^	9.4 × 10^10^	347	62.89	1.1 × 10^8^	4.8 × 10^10^	460

**Figure 3:**
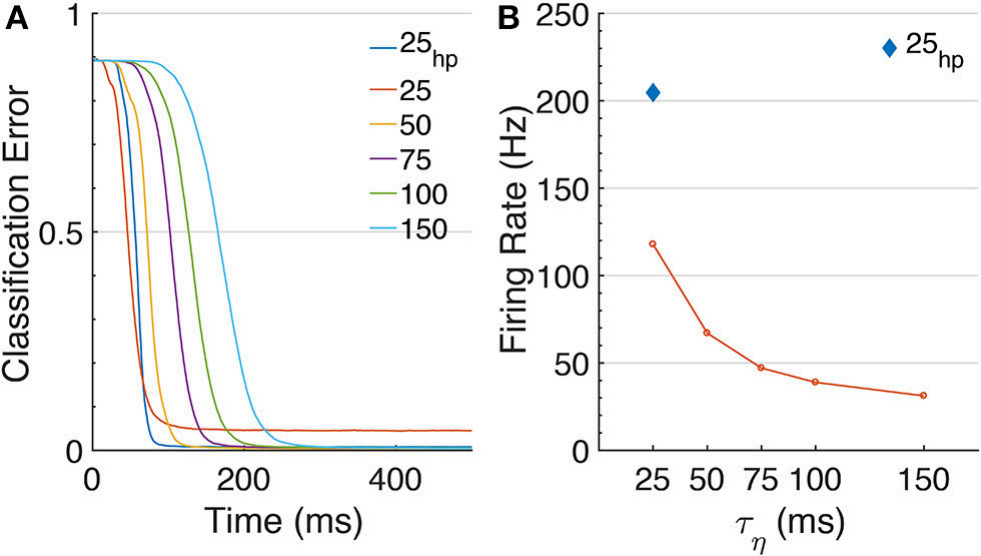
Effects of τ_η_ on MNIST. **(A)** The classification error over time is shown for increasing values of τ_η_s, [25, 50, 75, 100, 150] ms. Note that changing τ_η_ changes the transfer function shape, and thus different networks were trained. The plotted results are obtained with ϑ_0_ = 0.05. For τ_η_ = 25, we included an additional curve for higher precision at ϑ_0_ = 0.025, τ_η_ = 25_*hp*_. MT visibly increases for longer τ_η_s. **(B)** Networks' firing rates. Longer τ_η_s (and corresponding decay-times for the PSPs) require less spikes to approximate a signal.

We achieve this while requiring average firing-rates compatible with biological findings, in the range of 25-109 Hz; on some benchmarks, the AdSNNs exceed the ANNs performance, presumably because the AdSNNs compute an average from sampled neural activity (Hunsberger and Eliasmith, 2016) that correctly separates some additional input samples. As any SNN, the time-based communication in AdSNNs incurs latency, due to both the membrane filter ϕ and the adaptation process. We measure this as the time required between onset of the stimulus and the time when the output neurons are able to classify at the level of the network's analog counterpart. For AdSNNs, this latency (Matching Time, MT) is of order 300ms, and mainly depends on the PSP decay time (50 ms here); faster decay times result in lower latency, at the expense of increased firing rates (Figure 3A).

**Figure 4:**
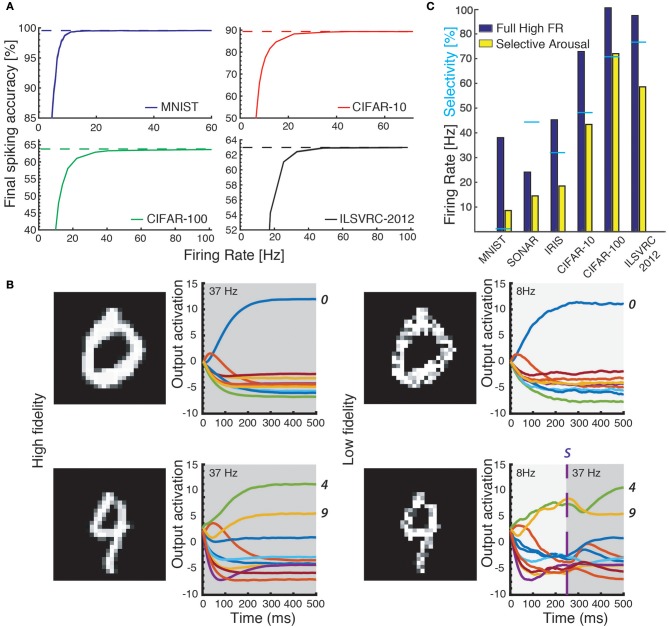
**(A)** Accuracy on the test sets of four datasets (MNIST, CIFAR-10 and CIFAR-100, ILSVRC-2012) as a function of average firing rate: accuracy decreases for lower coding precision (lower average firing rates in the network). **(B)** Classifying with attention. Ease of classification is measured as the distance between the internal value *S* of the winning output neuron and the second highest output neuron (line-plots). Top row: easy example that is correctly classified both at low precision (right) and high precision (left). Bottom row: ambiguous samples can be disambiguated by applying arousal to increase precision in the network. **(C)** The required number of spikes decreases further when Arousal is applied to hard-to-classify images only: the same classification accuracy is reached using a significantly lower average firing rate over the test set. Cyan bars designate percentage of inputs selected by the Arousal criterion: lower absolute performance results in higher selectivity and less benefit from Arousal.

We further find that AdSNNs exhibit a gradual and graceful performance degradation when the neural coding precision is decreased, by changing the ASN adaptation parameters such that the firing rate is lowered while increasing the effective spike height *h* (Figure 4A). Increasing the PSP decay time further lowers the required firing rate to achieve AdSNN performance matching ANNs at the expense of increased latency (Figure 3B).

**Figure 5:**
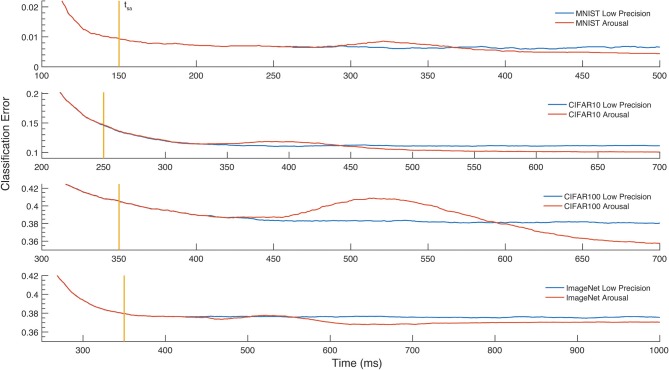
Classification error over time. The effect of the Arousal method on the classification error is reported for MNIST, CIFAR-10, CIFAR-100, and the Imagenet LSVRC-2012. The vertical line denotes the moment in time, *t*_*sa*_, where the outputs start being accumulated. Selection for Arousal is then determined 50ms later. The increase of the firing rate on selected images causes a brief loss of accuracy, after which a lower classification error is reached.

To exploit the tuneable relationship between firing rate and neural coding precision, we implement a simple attention model in the form of arousal affecting all neurons in the network simultaneously. Arousal is engaged selectively based on classification uncertainty: the neural coding precision is increased from a low base level only for samples deemed uncertain, as illustrated in Figure 4B. Uncertain inputs are identified by accumulating the two highest valued classification outputs for 50ms after a network-dependent fixed waiting time (dashed vertical line in Figure 4B). Arousal is engaged only if the averaged difference between these two outputs does not exceed a hard threshold as determined from the training set; engaging arousal causes a brief deterioration of classification accuracy before quickly settling to higher performance (Figure 5). Using this simple model of attentional modulation, the number of spikes required for overall classification is effectively halved (Figure 4C), while Matching Time latency increases as the selected inputs require additional time for classification (see Table 3). The uncertainty based arousal is also engaged more or less frequently depending on the accuracy of the model (blue markers in Figure 4C), and the benefit is thus greatest for networks with the highest absolute accuracy.

**Table 3:** Parameters used.

**DataSet**	**ϑ_0_**	**Selectivity (%)**	***t*_*sa*_ (ms)**	**ϑ_0−_*lp***	**ϑ_0−_*hp***	***p*_1_**	***p*_2_**
IRIS	0.08	32.00	150	0.40	0.08	2.2	−0.34 × 10^3^
SONAR	0.15	44.23	150	0.65	0.15	3.8	−0.52 × 10^3^
MNIST	0.05	1.13	200	0.30	0.060	5.6	−1.10 × 10^3^
CIFAR-10	0.025	48.07	250	0.075	0.025	9.4	−2.30 × 10^3^
CIFAR-100	0.025	70.50	350	0.05	0.015	12.0	−4.20 × 10^3^
LSVRC-2012	0.025	76.64	350	0.075	0.025	2.6	−0.84 × 10^3^

*The Arousal attention method as reported in Table 1 uses ϑ_0−lp_ by default and selectively switches to ϑ_0−hp_; ϑ_0_ denotes the resting threshold values used in the standard AdSNN*.

## Discussion

A number of recent studies have suggested that spiking neurons implement an efficient analog-to-digital conversion similar to the mechanisms proposed here (Lazar and Tóth, 2003; Boerlin and Denève, 2011; Bohte, 2012; Yoon, 2016). While population coding is a popular concept to explain how pools of spiking neurons can approximate analog signals with arbitrary precision (Denève and Machens, 2016), small nervous systems like the blow-fly do not have this luxury and single neurons are known to efficiently encode important quantities (Rieke et al., 1997). The results presented here show that firstly, the required neural coding precision in many deep neural networks can be satisfied with a single and plausible spiking neuron model at reasonable firing rates (tens of Herz, Hengen et al., 2013)—stochastic ASNs can similarly be used (Bohte, 2012) to increase biological plausibility though at considerable computational expense. Secondly, neural coding precision can further be increased or decreased by manipulating the firing rate inversely with a form of global synaptic efficacy modulation through the effective spike height *h*. This provides an alternative explanation for the observed attentional modulation of firing rates, and more detailed location-based or object-based attention algorithms can be studied to decrease the required number of spikes further.

As presented, the half-sigmoid-like derived transfer function we derived for ASNs holds for isomorphic spikes that can be communicated efficiently with a binary number. A rectified linear (ReLU) transfer function can be constructed by either tuning the weights and threshold to ensure that the spiking neuron mostly operates in a linear response regime in terms of input current and output firing rate, as in Diehl et al. (2015), with performance significantly removed from state-of-the-art in deep neural networks. Alternatively, a ReLU transfer function can be realized by scaling the impact of individual spikes on postsynaptic targets with the presynaptic adaptation magnitude at the time of spiking (Zambrano and Bohte, 2016; Chen et al., 2018). We find that using such a ReLU transfer function used in both ANN and SNN networks slightly improves performance and reduces latency, at the expense of communicating an analog rather than a binary value with each spike. From a biological perspective, such neural communication would require a tight coupling between neural adaptation and phenomena like synaptic facilitation and depression (Abbott and Regehr, 2004), which at present has not been examined in this context. From a computer science perspective, the efficiency penalty in terms of bandwidth may be limited as spike-based neuromorphic simulators like SpiNNaker already use sizable addressing bits for each spike (Furber et al., 2013); the computationally simple addition of spikes to the target neuron however is replaced by a conventional multiply-add operation.

AdSNNs explicitly use the time-dimension for communication and implicitly exploit temporal correlations in signals for sparse spike-based coding. In contrast, ANNs applied to temporal problem domains sequentially and synchronously sample their inputs in a time-stepped manner, recomputing the network for each successive timestep. This also applies to binarized networks (Courbariaux et al., 2016), where either weights or activations, or both, are constrained to binary values, but the entire networks is still recomputed for each timestep. Thus framed, binarized networks optimize a spatial version of network efficiency where AdSNNs aim to optimize temporal efficiency.

In terms of bandwidth, it takes the AdSNNs at most some 20 spikes per neuron to classify a CIFAR image, with a latency of 300ms, and hence 20 bits per neuron per image. It is hard to compare this number to efficient deep neural networks, but this number can serve as a starting point for comparing SNN architecture. The actual extraction of computational efficiency from sparsely active SNNs in implementations is a separate challenge. We find that while increasing the time-constant τ_η_ reduces the firing-rate further, this comes at the expense of response latency; for the classification of fixed stimuli, a network tuning approach like that in Diehl et al. (2015) could improve latency by setting weights and thresholds for individual neurons to negate the need for adaptation as much as possible.

The biology-inspired neural time-constants used in this work seem hard to reconcile with fast dynamics in recurrent neural networks. In a recent paper we demonstrated how a variant of an LSTM can be implemented with spiking neurons for cognitive tasks that involve working memory (Pozzi et al., 2018), this implementation however lacked recurrent connections. For fast dynamics, we may need to consider more complicated spiking neuron models like the iGIF model (Mensi et al., 2016) that incorporate the voltage-dependent interplay between AMPA and NMDA channels such that more active neurons use “faster” spikes (through shorter decay times). Alternatively, novel approaches for learning spatio-temporal patterns could alleviate the need for such recurrent networks to implement memory (Borovykh et al., 2017; Harczos and Klefenz, 2018).

In the presented model, sparse activity and computationally cheap connection updates are accompanied by a more complex and state-based neuron model that is updated more frequently. Networks with a high fan-in fan-out architecture, like the brain, benefit most from this trade-off; current deep learning architectures in contrast are characterized by a low degree of fan-in fan-out, except for the last layers which are typically fully connected. Hybrid analog/spiking neural network approaches may be most efficient for the implementation of these architectures. Additionally, similar to other state-based neural networks like LSTMs, and in contrast to feedforward ANN architectures, networks of adapting spiking neurons require per-neuron local memory to store state information such as potential and adaptation values. The availability of sufficient local memory is thus necessary to best extract efficiency from sparse spiking activity. Since current GPU-based deep learning accelerators are lacking in this regard, at least for the large state-based neural networks considered, neuromorphic digital hardware, such as the Intel Loihi chip (Davies et al., 2018), seems a promising approach for the implementation of large SNNs.

Concluding, our work suggests a novel way to approach spiking neuron models from sparse neural coding perspective, potentially linking to future neuroprosthetics and providing a framework to integrate unmodeled neuronal phenomena to improve coding efficiency, in particular in more dynamical settings.

## Data Availability Statement

The SONAR and IRIS datasets analyzed for this study can be found in the UCI Repository https://archive.ics.uci.edu/ml/datasets.html. The MNIST dataset is available at: http://yann.lecun.com/exdb/mnist/, and the LSVRC-2012 dataset at: http://image-net.org/challenges/LSVRC/2012/.

## Author Contributions

DZ and SB conceptualized the problem and the technical framework. RN derived the transfer function. HS, DZ, and SB developed the arousal method. DZ developed and tested the algorithms. DZ and SB wrote the manuscript with contributions from RN and HS. SB managed the project.

### Conflict of Interest Statement

The authors declare that the research was conducted in the absence of any commercial or financial relationships that could be construed as a potential conflict of interest.

## Footnotes

^1^https://keras.io/

^2^https://www.tensorflow.org/

^3^For MNIST, due to the low classification error we set the threshold to 120% of the minimum error (above 99.51% accuracy).

^4^http://www.vlfeat.org/matconvnet/

^5^The effective analog-to-spiking conversion approaches in Rueckauer et al. (2017) and Hunsberger and Eliasmith (2016) use a fully analog convolutional computation for the first layer of the network to avoid a significant performance drop from a Poisson-based conversion of inputs into spikes. Here, we directly encode inputs into spikes by using a first layer comprised of ASNs. The time-to-first-spike approach in Rueckauer and Liu (2018) is efficient in spikes but reverts to a frame-based processing paradigm.
