# Supplementary material for: Sparse Computation in Adaptive Spiking Neural Networks
Source: Front Neurosci. 2019 Jan 8;12:987. doi: 10.3389/fnins.2018.00987 (PMC6332470; doi:10.3389/fnins.2018.00987)
Supplement: Supplementary file 1 [file Data_Sheet_1.PDF]

Supplementary Material: Efficient Computation in Adaptive Spiking Neural Networks

1 SUPPLEMENTARY MATERIAL

To convert a trained Artificial Neural Network (ANN) into an Adaptive Spiking Neural Network (AdSNN), the transfer function of the ANN units needs to match the behaviour of the Adaptive Spiking Neuron (ASN). The ASN transfer function is derived for the general case of $\tau_\eta \neq \tau_\gamma$ using an approximation of the ASN behaviour.

Derivation of the ASN activation function

We consider a spiking neuron with activation $S(t)$ that is constant over time, and the refractory response $\hat{S}(t)$ approximates $S(t)$ using a variable threshold $\vartheta(t)$. Whenever $S - \hat{S}(t) > \vartheta(t)$, the neuron emits a spike of fixed height h to the synapses connecting to the target neurons, and a value of $2 \cdot \vartheta(t_f)$ is added to $\hat{S}$, with t_f the time of the spike. At the same time, the threshold is increased by $2 \cdot m_f \vartheta(t_f)/\vartheta_0$. The post-synaptic current (PSC) in the target neuron is then given by $I(t)$, which is convolved with the membrane filter $\phi(t)$ to obtain the contribution to the post-synaptic potential; a normalized exponential filter $\phi(t)$ with short time constants τ_ϕ smooths the high-frequency components of $I(t)$. We derive the transfer function that maps the activation S to the PSC I of the target neuron.

We recall the ASN model here, elaborating the SRM to include the current-to-potential filtering:

PSC:
$$I(t) = \sum_i \sum_{t_f^i} w_i \exp\left(\frac{t_f^i - t}{\tau_\beta}\right), \tag{S1}$$

activation:
$$\begin{aligned} S(t) &= (\phi * I)(t) \\ &= \sum_i \sum_{t_f^i} w_i \kappa\left(t - t_f^i\right), \end{aligned} \tag{S2}$$

threshold:
$$\begin{aligned} \vartheta(t) &= \vartheta_0 + \sum_{t_f} \frac{m_f}{\vartheta_0} \vartheta(t_f) 2 \exp\left(\frac{t_f - t}{\tau_\gamma}\right) \\ &= \sum_{t_f} \frac{m_f}{\vartheta_0} \vartheta(t_f) \gamma\left(t - t_f\right), \end{aligned} \tag{S3}$$

refractory response:
$$\begin{aligned} \hat{S}(t) &= \sum_{t_f} \vartheta(t_f) 2 \exp\left(\frac{t_f - t}{\tau_\eta}\right) \\ &= \sum_{t_f} \vartheta(t_f) \eta\left(t - t_f\right), \end{aligned} \tag{S4}$$

where t_f^i denotes the timing of incoming spikes that the neuron receives and t_f the timing of outgoing spikes.

Since the variables of the ASN decay exponentially, they converge asymptotically. For a given fixed size current injection, we consider a neuron that has stabilised around an equilibrium, that is $\hat{S}(t)$ and $\vartheta(t)$ at the time of a spike always reach the same values. Let these values be denoted as $\hat{S}_l$ and ϑ_l respectively. Then, $\vartheta(t_f) = \vartheta_l$ and $\hat{S}(t_f) = \hat{S}_l$ for all t_f. The PSC $I(t)$ also always declines to the same value, I_l, before it receives a new spike. Setting $t = 0$ for the last time that there was a spike, we can rewrite our ASN equations, Equations (S1), (S2), (S3) and (S4), for $\tau_\beta = \tau_\eta$ and $0 < t < t_f$ to:

$$\hat{S}(t) = \hat{S}_l e^{-\frac{t}{\tau_\eta}} + 2 \cdot \vartheta_l e^{-\frac{t}{\tau_\eta}}, \tag{S5}$$

$$\vartheta(t) = \vartheta_0 + (\vartheta_l - \vartheta_0) e^{-\frac{t}{\tau_\gamma}} + 2 \cdot \frac{m_f}{\vartheta_0} \vartheta_l e^{-\frac{t}{\tau_\gamma}}, \tag{S6}$$

$$I(t) = I_l e^{-\frac{t}{\tau_\eta}} + h e^{-\frac{t}{\tau_\eta}}. \tag{S7}$$

The transfer function $f(S)$ of the ASN is a function of the value of S; $f(S)$ should be a bit larger than I_l since that is the lowest value of $I(t)$, and we are interested in the average value of $I(t)$ between two spikes: $f(S) = I_{average}$.

Since we are in a stable situation, the time between each spike is fixed; we define this time as t_e. Thus, if the last spike occurred at $t = 0$, the next spike should happen at $t = t_e$. This implies that $\hat{S}(t), \vartheta(t)$ and $I(t)$ at $t = t_e$ must have reached their minimal values $\hat{S}_l, \vartheta_l$ and I_l respectively.

To obtain the activation function $f(S)$, we solve the following set of equations:

$$\hat{S}(t_e) = \hat{S}_l,$$
$$\vartheta(t_e) = \vartheta_l,$$
$$I(t_e) = I_l,$$

and by noting that the neuron only emits a spike when $S - \hat{S}(t) > \vartheta$, we also have:

$$S - \hat{S}_l = \vartheta_l.$$

We first notice:

$$\frac{h e^{-\frac{t_e}{\tau_\eta}}}{1 - e^{-\frac{t_e}{\tau_\eta}}} = I_l. \tag{S8}$$

We now want an expression for ϑ_l:

$$\vartheta_0 + (\vartheta_l - \vartheta_0)e^{-\frac{t_e}{\tau_\gamma}} + 2 \cdot \frac{m_f}{\vartheta_0}\vartheta_l e^{-\frac{t_e}{\tau_\gamma}} = \vartheta_l,$$
$$\vartheta_0 - \vartheta_0 e^{-\frac{t_e}{\tau_\gamma}} = \vartheta_l - 2 \cdot \frac{m_f}{\vartheta_0}\vartheta_l e^{-\frac{t_e}{\tau_\gamma}} - \vartheta_l e^{-\frac{t_e}{\tau_\gamma}}.$$

We can rewrite this to:

$$\vartheta_0 \frac{1 - e^{-\frac{t_e}{\tau_\gamma}}}{1 - (2 \cdot \frac{m_f}{\vartheta_0} + 1)e^{-\frac{t_e}{\tau_\gamma}}} = \vartheta_l. \quad \text{(S9)}$$

Using equations $S - \hat{S}_l = \vartheta_l$ and $\hat{S}(t_e) = \hat{S}_l$, we get:

$$(S - \vartheta_l)e^{-\frac{t_e}{\tau_\eta}} + 2 \cdot \vartheta_l e^{-\frac{t_e}{\tau_\eta}} = S - \vartheta_l,$$
$$e^{-\frac{t_e}{\tau_\eta}}(S + \vartheta_l) = S - \vartheta_l.$$

Inserting Equation S9 gives:

$$e^{-\frac{t_e}{\tau_\eta}} \left(S + \vartheta_0 \frac{1 - e^{-\frac{t_e}{\tau_\gamma}}}{1 - (2 \cdot \frac{m_f}{\vartheta_0} + 1)e^{-\frac{t_e}{\tau_\gamma}}} \right)$$
$$= S - \vartheta_0 \frac{1 - e^{-\frac{t_e}{\tau_\gamma}}}{1 - (2 \cdot \frac{m_f}{\vartheta_0} + 1)e^{-\frac{t_e}{\tau_\gamma}}}.$$

This can be rewritten to:

$$e^{-\frac{t_e}{\tau_\eta}} \left(S(1 - (2 \cdot \frac{m_f}{\vartheta_0} + 1)e^{-\frac{t_e}{\tau_\gamma}}) + \vartheta_0(1 - e^{-\frac{t_e}{\tau_\gamma}}) \right)$$
$$= S(1 - (2 \cdot \frac{m_f}{\vartheta_0} + 1)e^{-\frac{t_e}{\tau_\gamma}}) - \vartheta_0(1 - e^{-\frac{t_e}{\tau_\gamma}}),$$
$$(S + \vartheta_0)e^{-\frac{t_e}{\tau_\eta}} - (S(2 \cdot \frac{m_f}{\vartheta_0} + 1) + \vartheta_0)e^{-\frac{t_e}{\tau_\gamma}}e^{-\frac{t_e}{\tau_\eta}}$$
$$= S - \vartheta_0 - S(2 \cdot \frac{m_f}{\vartheta_0} + 1)e^{-\frac{t_e}{\tau_\gamma}} + \vartheta_0 e^{-\frac{t_e}{\tau_\gamma}},$$
$$(S + \vartheta_0)e^{-\frac{t_e}{\tau_\eta}} - (S(2 \cdot \frac{m_f}{\vartheta_0} + 1) + \vartheta_0)e^{-t_e(\frac{1}{\tau_\gamma} + \frac{1}{\tau_\eta})}$$
$$+ (S(2 \cdot \frac{m_f}{\vartheta_0} + 1) - \vartheta_0)e^{-\frac{t_e}{\tau_\gamma}} = S - \vartheta_0. \quad \text{(S10)}$$

Approximation of the AAN activation function

In the general case of $\tau_\eta \neq \tau_\gamma$, a (second order) Taylor series expansion can be used to approximate the exponential function:

$$e^x \approx 1 + x + \frac{x^2}{2},$$

for x close to 0. We can use this in our previous equation:

$$\begin{aligned}(S+\vartheta_0)(1-\frac{1}{\tau_\eta}t_e+\frac{1}{2\tau_\eta^2}t_e^2)-(S(2\cdot\frac{m_f}{\vartheta_0}+1)+\vartheta_0)\\(1-(\frac{1}{\tau_\gamma}+\frac{1}{\tau_\eta})t_e+\frac{1}{2}(\frac{1}{\tau_\gamma}+\frac{1}{\tau_\eta})^2t_e^2)\\+(S(2\cdot\frac{m_f}{\vartheta_0}+1)-\vartheta_0)(1-\frac{1}{\tau_\gamma}t_e+\frac{1}{2\tau_\gamma^2}t_e^2)\\=S-\vartheta_0.\end{aligned}$$

We need a few steps to isolate t_e:

$$\begin{aligned}(S+\vartheta_0)(-\frac{1}{\tau_\eta}t_e+\frac{1}{2\tau_\eta^2}t_e^2)-(S(2\cdot\frac{m_f}{\vartheta_0}+1)+\vartheta_0)\\(-(\frac{1}{\tau_\gamma}+\frac{1}{\tau_\eta})t_e+\frac{1}{2}(\frac{1}{\tau_\gamma}+\frac{1}{\tau_\eta})^2t_e^2)\\+(S(2\cdot\frac{m_f}{\vartheta_0}+1)-\vartheta_0)(-\frac{1}{\tau_\gamma}t_e+\frac{1}{2\tau_\gamma^2}t_e^2)=0,\\(S+\vartheta_0)(-\frac{1}{\tau_\eta}+\frac{1}{2\tau_\eta^2}t_e)-(S(2\cdot\frac{m_f}{\vartheta_0}+1)+\vartheta_0)\\(-(\frac{1}{\tau_\gamma}+\frac{1}{\tau_\eta})+\frac{1}{2}(\frac{1}{\tau_\gamma}+\frac{1}{\tau_\eta})^2t_e)\\+(S(2\cdot\frac{m_f}{\vartheta_0}+1)-\vartheta_0)(-\frac{1}{\tau_\gamma}+\frac{1}{2\tau_\gamma^2}t_e)=0,\end{aligned}$$

$$((S+\vartheta_0)\frac{1}{2\tau_\eta^2}-(S(2\cdot\frac{m_f}{\vartheta_0}+1)+\vartheta_0)\frac{1}{2}(\frac{1}{\tau_\gamma}+\frac{1}{\tau_\eta})^2$$
$$+(S(2\cdot\frac{m_f}{\vartheta_0}+1)-\vartheta_0)\frac{1}{2\tau_\gamma^2})t_e$$
$$=(S+\vartheta_0)\frac{1}{\tau_\eta}-(S(2\cdot\frac{m_f}{\vartheta_0}+1)+\vartheta_0)(\frac{1}{\tau_\gamma}+\frac{1}{\tau_\eta})$$
$$+(S(2\cdot\frac{m_f}{\vartheta_0}+1)-\vartheta_0)\frac{1}{\tau_\gamma},$$

$$(-S\cdot\frac{m_f}{\vartheta_0}\frac{1}{\tau_\eta^2}-(S(2\cdot\frac{m_f}{\vartheta_0}+1)+\vartheta_0)\frac{1}{\tau_\gamma\tau_\eta}-\vartheta_0\frac{1}{\tau_\gamma^2})t_e$$
$$=-S\cdot\frac{m_f}{\vartheta_0}\frac{1}{\tau_\eta}-2\vartheta_0\frac{1}{\tau_\gamma},$$
$$(-S\cdot\frac{m_f}{\vartheta_0}\tau_\gamma^2-(S(2\cdot\frac{m_f}{\vartheta_0}+1)+\vartheta_0)\tau_\gamma\tau_\eta-\vartheta_0\tau_\eta^2)t_e$$
$$=-2S\cdot\frac{m_f}{\vartheta_0}\tau_\gamma^2\tau_\eta-2\vartheta_0\tau_\gamma\tau_\eta^2.$$

This leads to our expression for t_e :

$$t_e=\frac{2\cdot\tau_\gamma\tau_\eta(S\cdot\frac{m_f}{\vartheta_0}\tau_\gamma+\cdot\vartheta_0\tau_\eta)}{S\cdot\tau_\gamma(\frac{m_f}{\vartheta_0}\tau_\gamma+(2\cdot\frac{m_f}{\vartheta_0}+1)\tau_\eta)+\vartheta_0\tau_\gamma\tau_\eta+\vartheta_0\tau_\eta^2}.$$

We now insert this expression in Equation S8 and get:

$$I_l(S)=\frac{h}{e^{\frac{t_e}{\tau_\eta}}-1}=$$
$$\frac{h}{\exp\left(\frac{2\tau_\gamma(S\cdot\frac{m_f}{\vartheta_0}\tau_\gamma+\vartheta_0\tau_\eta)}{S\cdot\tau_\gamma(\frac{m_f}{\vartheta_0}\tau_\gamma+(2\cdot\frac{m_f}{\vartheta_0}+1)\tau_\eta)+\vartheta_0\tau_\gamma\tau_\eta+\vartheta_0\tau_\eta^2}\right)-1}.$$

To make sure that our activation function $f(S)$ is 0 at $S=\vartheta_0$ we choose our activation function to be:

$$f(S)=I_l(S)-I_l(\vartheta_0)=$$
$$\frac{h}{\exp\left(\frac{2\frac{m_f}{\vartheta_0}\tau_\gamma^2S+2\vartheta_0\tau_\eta\tau_\gamma}{\tau_\gamma(\frac{m_f}{\vartheta_0}\tau_\gamma+(2\cdot\frac{m_f}{\vartheta_0}+1)\tau_\eta)S+\vartheta_0\tau_\gamma\tau_\eta+\vartheta_0\tau_\eta^2}\right)-1}-c, \tag{S11}$$

for $S>\vartheta_0$ and $f(S)=0$ for $S<=\vartheta_0$ with $c=I_l(\vartheta_0)$.

2 SUPPLEMENTARY FIGURE

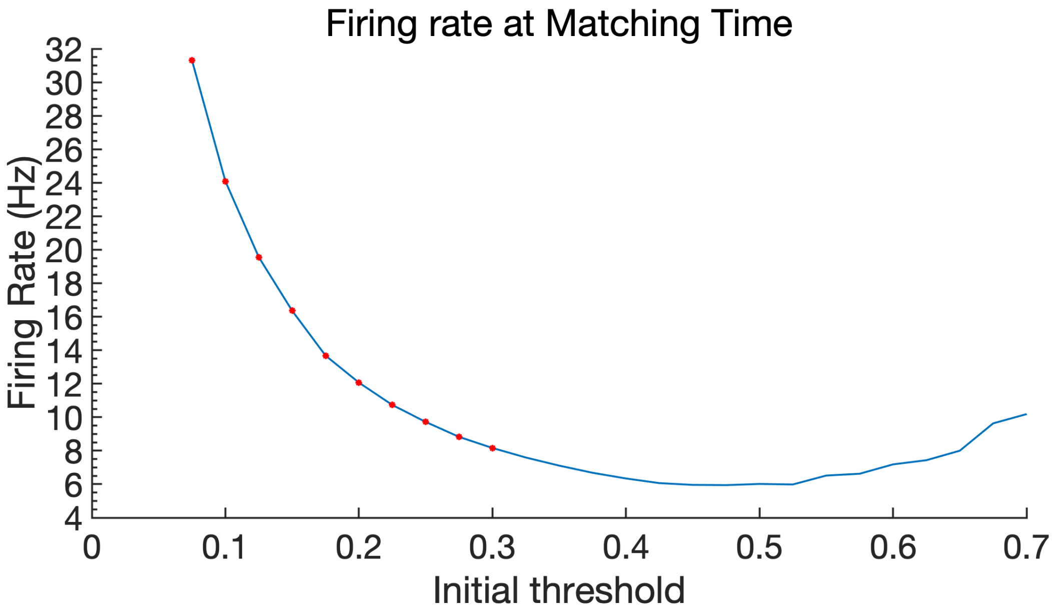

Figure S1. Firing Rate (Hz) at different initial thresholds (ϑ_{0-lp}) for the Arousal method for MNIST. The graph shows the Firing Rate computed as the number of spikes emitted until MT with different initial thresholds (ϑ_{0-lp}, low-precision). Starting with either high or low ϑ_{0-lp} yields to a high number of emitted spikes. The red-dots represent the ϑ_{0-lp} values for which the final accuracy is effectively matched within the considered time window (see the definition of MT in the main text). ϑ_{0-lp} has then been chosen as the value that minimises the final Firing Rate.
